# A participatory approach to elucidate the consequences of land invasions on REDD+ initiatives: A case study with Indigenous communities in Panama

**DOI:** 10.1371/journal.pone.0189463

**Published:** 2017-12-19

**Authors:** Gerardo Vergara-Asenjo, Javier Mateo-Vega, Alexis Alvarado, Catherine Potvin

**Affiliations:** 1 Department of Biology, McGill University, Montreal, Quebec, Canada; 2 Forest Research Institute (INFOR), Valdivia, Chile; 3 Smithsonian Tropical Research Institute, Panama City, Republic of Panama; 4 Fundación Dobbo Yala, Panama City, Republic of Panama; Missouri Botanical Garden, UNITED STATES

## Abstract

Land tenure and tenure security are among the most important factors determining the viability and success of Reducing Emissions from Deforestation and Forest Degradation (REDD+) initiatives. The premise of the present paper is that territorial conflicts lead to forest loss and compromise the successful implementation of REDD+. Within this context, the main objectives of this paper are to (i) document, relying on participatory methods, the extent to which land conflicts drive deforestation and (ii) reflect on the legal context of REDD+ examining if, from an Indigenous perspective, it offers tools to resolve such conflicts. We used the Upper Bayano Watershed in eastern Panama as a case study of complex land tenure dynamics, and their effects on forest conservation in the context of REDD+. Combining a range of participatory methods including participatory mapping and forest carbon stock assessment, we estimated the consequences of land invasions on forest carbon stocks. Our analysis shows that invasions of Indigenous territories amounted to 27.6% of the total deforestation for the period of 2001–2014. The situation is of paramount concern in the Embera territory of Majé where 95.4% of total deforestation was caused by colonist invaders. Using and validating the maps made freely available by the Global Forest Change initiative of the University of Maryland, we then developed a reference level for the watershed and carried out a back of the envelop estimation of likely REDD+ revenue, showing its potential to bring much needed income to Indigenous communities striving to protect their forest estate. Our analysis of current legislation in Panama highlights confusion and important legal voids and emphasizes the strong links between land tenure, carbon ownership, and territorial invasions. The options and shortcoming of implementing REDD+ in Indigenous territories is discussed in the conclusion taking our legal review into account.

## Introduction

Tenure security–defined as the certainty that a community’s land rights will be recognized and protected if challenged [1]–is one of the prevailing influences on deforestation, forest degradation and the expansion of extensive cattle ranching, which are among the primary drivers of land use change in Latin America [2–4]. Furthermore, land tenure defines who is eligible to receive REDD+ benefits, and influences the ability of recipients to enforce carbon contracts [5]. Therefore, land tenure and carbon ownership rights are intimately linked with land tenure security, which is critical in achieving successful emissions reductions through REDD+ [6–9].

This study is part of a long-term initiative that began in the early 2000s to examine if and how payment for ecosystem services, such as the Clean Development Mechanism or REDD+, may contribute toward improving local livelihoods while preserving forest carbon stocks in Panama [10–13]. Concerned that REDD+ might ignite or compound territorial conflicts between forest stewards and others (*e*.*g*. landless farmers, land speculators, loggers, and cattle ranchers) trying to gain control over land or potentially lucrative forest resources, we developed the project, “*Establishing a novel intercultural partnership for REDD*+” (hereafter, the *Partnership Project*), between 2010 and 2013 in collaboration with the *Dobbo Yala Foundation*, a Panama-based, non-governmental organization (NGO) comprised of Indigenous professionals (directed by co-author and lawyer, AA), which works with communities and traditional authorities in defending Indigenous rights and environmental integrity; the *National Coordinating Body of Panama’s Indigenous Peoples (Coordinadora Nacional de los Pueblos Indígenas de Panamá*, *COONAPIP in Spanish)* that brings together all seven Indigenous Nations and their twelve Congresses; and *Panama’s Embera and Wounaan Youth Organization (Organización de Jóvenes Emberá y Wounaan de Panamá*, *OJEWP in Spanish*), an NGO that fights for the rights of the Emberá and Wounaan, particularly youth, and provides technical support to traditional authorities; as well as the *Center for the Study and Resolution of Conflicts in the Americas and the Caribbean (Centro de Estudios y Resolución de Conflictos de las Américas*, *CERCA in Spanish)*, an NGO that promotes alternative methods of conflict resolution in the region. Through this project, we examined factors that could support ongoing conservation efforts by Indigenous peoples, while clarifying, from an Indigenous perspective, the legislative context of REDD+, with a view toward identifying perverse incentives or barriers to implementation, and tools to support action.

**Fig 1:**
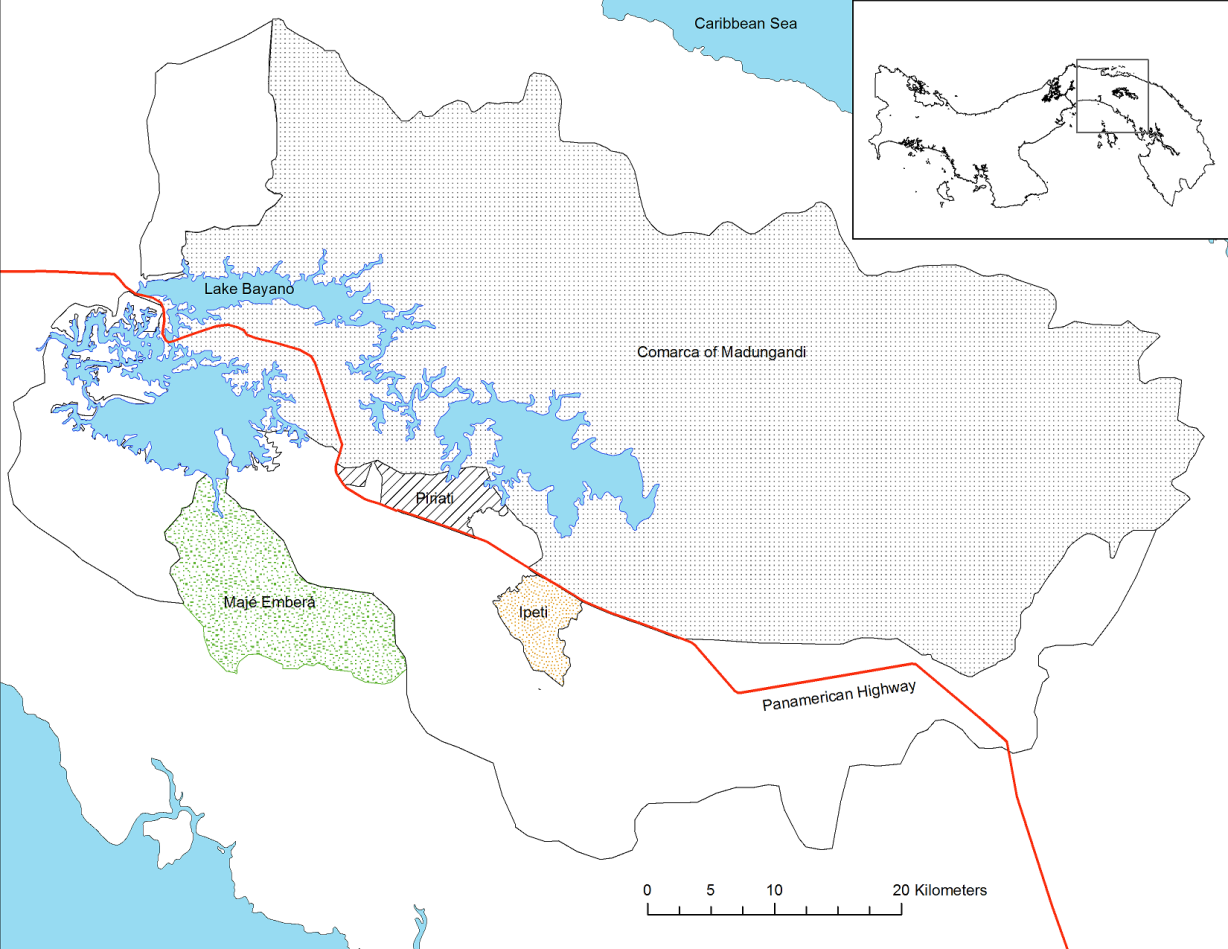
The Indigenous territories in the Upper Bayano Watershed in eastern Panama.

The premise of the present paper is that territorial conflicts lead to forest loss and compromise the successful implementation of REDD+. Within this context, the main objectives of this study are to (i) document, relying on participatory methods, the extent to which land conflicts drive deforestation and (ii) reflect on the legal context of REDD+ if, from an Indigenous perspective, it offers tools to resolve such conflicts. We focus on the Upper Bayano Watershed (Fig 1) a 3,695-km^2^ region inhabited by Guna and Embera Indigenous peoples and colonist farmers of Latino origin that has been subject to complex land tenure dynamics [14], particularly since 1976, when low-lying areas of eastern Panama were flooded to create the 350 km^2^ artificial Lake Bayano. The flooding forced Indigenous peoples to resettle in other parts of the watershed. The impacts of resettlement on the Guna have been aptly portrayed in a moving documentary produced by Guna and Embera youth in 2014 (Text A in S1 File). Since resettlement, the watershed has been plagued by invasions of colonist farmers in Indigenous territories [15]. Colonist farmers largely see forested Indigenous territories as “free and unexploited” lands, therefore justifying their invasions of these areas [16]. In 2014, the Inter-American Court of Human Rights (IACHR) established jurisprudence on Indigenous territorial rights internationally by ruling in favor of the Gunas and Emberas of the Upper Bayano Watershed in their case against the Government of Panama [17]. The IACHR mandated the Government of Panama, which had been, for the most part, unwilling or unable to resolve land use conflicts in the region, to address land tenure and tenure security.

## Methods

### Locating and mapping invasion areas

**Fig 2:**
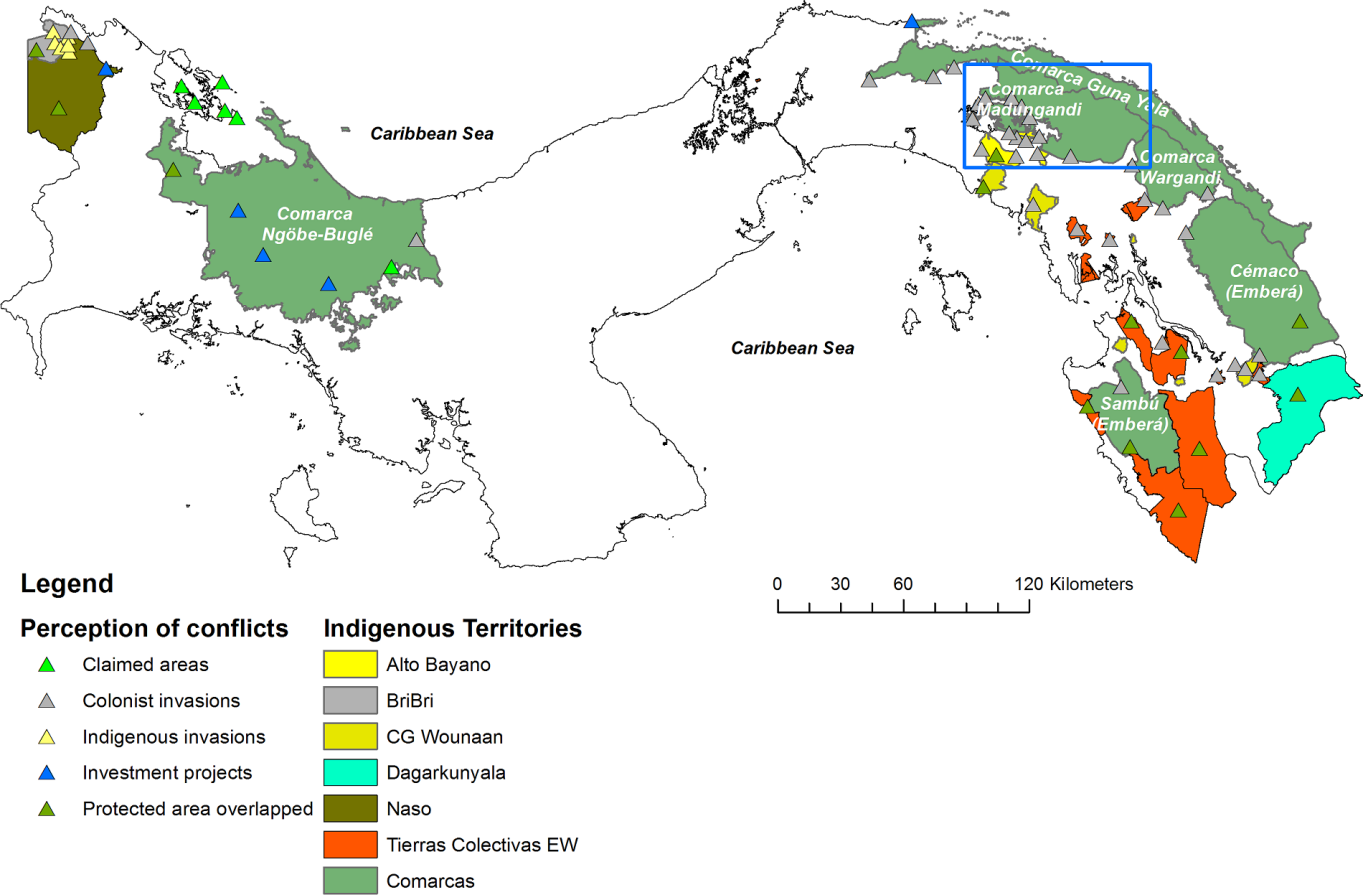
Map of territorial and resource conflicts in Indigenous lands in Panama. Typology and distribution of territorial conflicts in Indigenous territories according to the perception of Indigenous communities in Panama.

In 2011, COONAPIP asked co-author, GVA, to map the country’s Indigenous territories [13], as well as the location of territorial and natural resource conflicts in these territories. For this, Indigenous leaders were interviewed and information was collected from government institutions. The result was the creation of a map of Indigenous peoples perceptions of territorial conflicts. Traditional authorities and technicians from ten of the 12 general Indigenous congresses validated the map in a workshop held in the Comarca Ngäbe Bugle in August 2011. Different conflict categories such as land invasions, investment projects (*e*.*g*. hydroelectric dams), and territories overlapping with protected areas, among others, were identified (Fig 2).

This work revealed an important number of land invasions in the Upper Bayano Watershed. As a result, twenty informal interviews were subsequently held with Indigenous leaders and government technicians in the region, which allowed us to further document current and unresolved land invasions in the watershed. Finally, community members built a three-dimensional scale model of the watershed, identifying areas of conflict and helping us to develop a timeline of land invasions [18]. Validation of all the information obtained on the invaded areas was then carried out with Indigenous leaders and technicians over the course of three workshops, one with the Guna and two with the Embera, in November 2015 attended by a total of 25 people. Finally, Indigenous leaders and technicians, as well as co-author GVA, visited three specific areas identified as invasion sites after the final workshop, because participants felt that the boundaries of these particular invasions needed to be clarified in the field.

### Estimating the impacts of land invasions on forests

#### Study area

To fulfill our objective of estimating the link between deforestation and territorial conflicts, we developed a reference level of forest carbon emissions offsets [19] for the Upper Bayano Watershed (Fig 1) while maximising stakeholder engagement. The United Nations Framework Convention on Climate Change (UNFCCC), indeed calls for the full and effective participation of local and Indigenous peoples (Decision 89 4/CP. 15 and Decision 1/CP. 16 [20]) in the measuring, reporting and verifying (MRV) requirements for REDD+. We combined several participatory methods building on previous studies that demonstrated the accuracy of participatory mapping [21] and forest biomass estimation [22].

Within the Upper Bayano Watershed, Guna people inhabit the Comarca Madungandi, officially recognized by the Panamanian Government under Law 24 of January 12, 1996. It is the territory with the longest Indigenous tenure in the watershed. Emberas inhabit the Collective Lands (clusters of Indigenous territories that are in process of being adjudicated and titled) of Alto Bayano consisting of three separate areas–*i*.*e*. Ipeti, Piriati and Majé –in the southern portion of the watershed. Piriati and Ipeti were legally recognized by the Panamanian government under the country’s Law 72 [23] in 2014 and 2015, respectively, after a long legal dispute. Land title has not yet been recognized for Majé.

The gap-filled Shuttle Radar Topography Mission (SRTM) digital elevation model (DEM) version 4 [24] at 90 m resolution, from the CGIAR Consortium for Spatial Information (http://srtm.csi.cgiar.org/), was used to delineate the watershed boundaries. According to the Holdridge Life Zone system, the watershed is primarily covered by “tropical moist”, “premontane wet” and “tropical wet” forests [25]. Elevation in the watershed ranges from 60 to 1,080 m above sea level, with Majé housing the areas with highest elevation. Average annual precipitation ranges between 2,000 and 3,000 mm. Annual temperature averages 26°C in the lowlands and 22°C in highlands, with a pronounced dry season from December to April [26].

#### Land use change data collection, analysis and validation

To understand the dynamics of land cover and land-use change in the study area, we collected information on the extension of Indigenous territories and analyzed historical documents from government and NGOs that included descriptions, locations and other relevant details about land use conflicts. These sources were complemented with documents provided to us by Indigenous organizations.

We chose to work with the Hansen et al. [27] forest cover change maps, which are the first global, wall-to-wall, annual forest cover change maps freely available online. They use time-series analysis of Landsat images at 30x30m spatial resolution that can be translated to carbon dioxide emissions for REDD+ accounting if combined with emissions factors derived for the assessment of forest carbon stocks. We felt that the Hansen et al. [27] maps would allow communities and groups of stakeholders without the financial means to pay for satellite imagery, or without the technical capacity to analyse remote sensing images, to build reference levels of deforestation. Local technicians verified the potential for the Hansen et al. [27] maps to develop REDD+ reference level for the Upper Bayano Watershed as described below.

We began by organizing in 2014, a mapping and GIS training course using Nexus 7 tablets, donated by Google through their program, *Androids for Good*. Over the course of eight working sessions, led by Dr. J. Pablo Arroyo-Mora (then Director of McGill University’s Geographic Information Centre), participants learned to use the tablets and software. Approximately 40 Guna and Embera Indigenous technicians, local farmers and local students of the University of Panama’s Certificate Program on Watershed Management, learned to use five different software packages including Global Positioning Systems (GPS) and Geographic Information Systems (GIS) software such as ODK Collect to conduct surveys, GPStest and GPSEssential to mark and register points and areas, and Planimeter and QGIS to map, analyze and manage the spatially explicit data that they gathered. This course was conceived to help participants develop autonomy in (1) marking the limits of their territories; (2) registering the location of forested areas; (3) documenting land invasions, and areas being actively deforested or degraded; and (4) mapping sites vulnerable to extreme natural events, such a flood-prone areas. The workshops included both classroom and field-based exercises. During the workshops, participants became familiar with the maps produced by Hansen et al. [27] thanks to the web-based versions publically available on the website of Global Forest Watch (http://www.globalforestwatch.org). The Embera and Guna Indigenous authorities and technicians used the tablets and skills learned to carry out multiple field trips to map and verify the limits of their territories and document land invasions.

**Fig 3:**
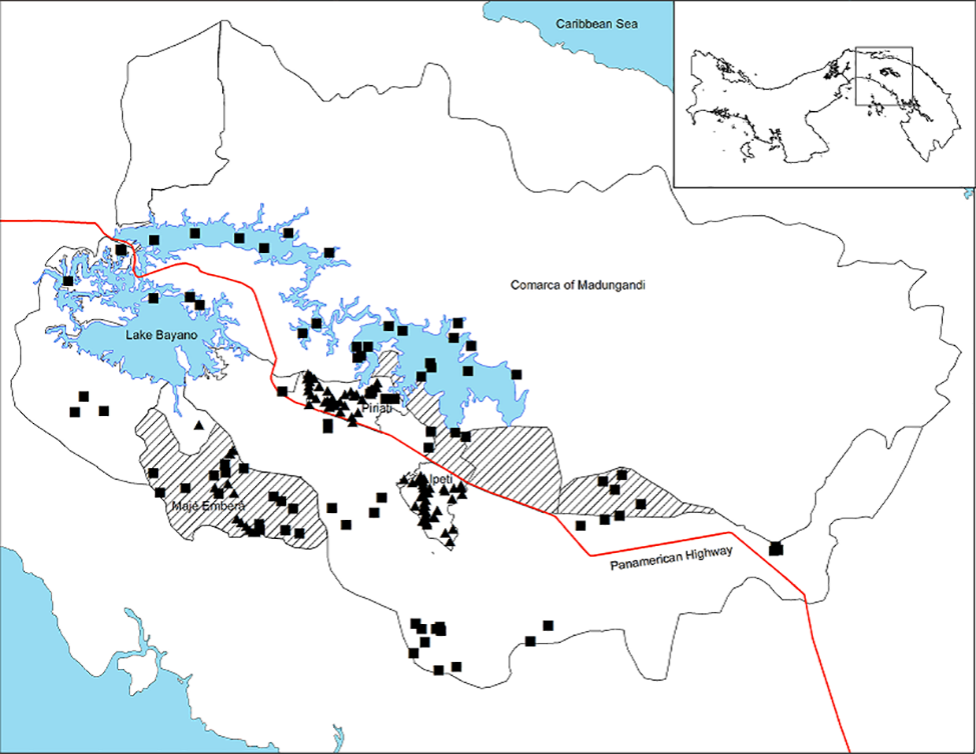
Map of ground truthing points in the Upper Bayano Watershed. Participatory ground truthing data of the Hansen et al. [27] maps in the Upper Bayano. Forest inventory data is represented with triangles and validation points are represented with squares. Invaded areas are identified with hatched patterns.

Once the Indigenous technicians had familiarized themselves with the use of the GPS, and the relationship between geographic position and mapping, we set out to validate the accuracy of the Hansen et al. [27] maps for the watershed. The Hansen et al. [27] global forest cover and forest cover change classification maps (encoded in binary format, with 1 as loss or 0 as no loss) are derived from Landsat imagery for the period 2000–2014 using multispectral satellite imagery from the Landsat 7 thematic mapper plus (ETM+), and Landsat 8 Operational Land Imager (OLI) sensors. To evaluate the local accuracy of the maps produced by Hansen et al. [27] in estimating deforestation in the watershed, 173 randomly selected control points were used to verify land cover in the field by 12 Embera and Guna Indigenous technicians supervised by scientists from our research group, the Neotropical Ecology Laboratory (NEL, McGill University and Smithsonian Tropical Research Institute). Land cover at each control point was verified in the field using a Garmin GPSMAP 60CSx by the local Indigenous technicians previously trained in operating GPS devices and identifying current land cover. The technicians carried out informal consultations with local inhabitants, when possible, to enquire about land conversion dates. Two sets of control points were used to validate forest cover and forest cover change in 2013 and 2014 (Fig 3). The first set consisted of 90 forest inventory plots established by the Indigenous technicians in 2012–2013 across the watershed in areas representing a range of elevational and human intervention gradients [21]. The second set consisted of an additional 83 control points that were visited by the Indigenous technicians to assess the accuracy of the classification of forest cover change. Thirty of these points corresponded to water or shallow waters, as the Hansen et al. [27] maps appear to have identified some areas in shallow waters around Lake Bayano as forest cover or forest cover change. The remaining 53 random points in forest cover change areas were restricted to changes that occurred in 2013 and 2014 (21 and 32 points respectively) easily accessible in the field.

Together, the 173 validation points served to verify forest classification, as well as forest cover change in the study area. The accuracy classification of the Hansen et al. [27] maps was estimated using a confusion matrix [21, 28], which calculates a classification error indicating the discrepancy between the situation depicted on the map and the reality observed in the field [29]. Producer and user accuracies–two measures of omission and commission errors, respectively–were also estimated to obtain an assessment of how well specific land categories in the Hansen et al. [27] maps were classified. The producer’s accuracy refers to the probability that the land-cover of a certain area on the ground is classified as such, while the user’s accuracy refers to the probability that a pixel labeled as a certain land-cover class in the map is, in fact, the class.

#### Reference emission levels

To estimate emission factors, we established and inventoried forest plots with Indigenous technicians under the supervision of NEL scientists. The technicians received both theoretical and hands-on training during a three-day workshop, which included learning the use of forest instruments under different conditions of vegetation and terrain, *i*.*e*. slope correction. Our research group began involving Indigenous technicians in forest carbon measurements as early as 2004 [30]. Taking advantage of these existing skillsets, each field crew was comprised of groups of three Embera technicians, led by at least one experienced technician. For the present study, we used a set of 38 forest inventory plots established in the three Embera territories which are also representatives of the vegetation types of the Comarca Madungandi (S2 File). Trees >25 cm DBH were measured in 25 m-radius plot, while all trees > 10 cm DBH were measured in a nested 6 m- radius subplot. Tree height, DBH and species identification were recorded.

Above ground biomass (AGB) was estimated using allometric equations produced by Chave et al. [31] with wood density values taken from the database created by Wright et al. [32]. AGB was estimated for each individual tree and then summed up at plot level. AGB for smaller stems (*i*.*e*. ≥10cm—<25cm DBH) sampled in the 6m-radius subplots were applied a conversion factor to account for the entire 25m-radius plot. For emissions factors, we calculated an average AGB per hectare using a single factor according to each ecozone (Holdrigde life zones) in the study area. Average AGB per ecozone was then transformed to carbon fraction using a factor of 0.47 [33] and then converted to ton of carbon dioxide equivalent (tCO_2_e) using the standard conversion factor of 3.67. Total CO_2_ emissions produced by deforestation between 2001 and 2014 were estimated multiplying the average value of carbon dioxide equivalent per ecozone with the deforested areas identified in the Hansen et al. [27] maps. To do so, we overlaid a Holdridge life ecozone layer on the Hansen et al. [27] maps of land cover change. Deforestation due to invasions was differentiated from other causes to determine its impact on the watershed.

A predictive analysis of deforestation was then carried out using the Land Cover Change (LCM) module of the Terrset software [34] for the period 2015–2024. Slope, elevation, distance from previous deforestation and distance from roads were included as potential drivers in the model. The prediction process was based on Markov model using a “hard” predictor [34]. The model assumes no improvement in governance and a continued pattern of land invasions. Land change prediction in Terrset´s Land Change Modeler (LCM) is an empirically-driven process that moves in a stepwise fashion from 1) Change Analysis, to 2) Transition Potential Modeling, to 3) Change Prediction. It is based on the historical change from time 1 to time 2 of land cover maps to project future scenarios. A “hard” prediction model projects future land cover of each pixel based on the model's drivers (slope, etc.) but considers only one possible outcome. As such, the hard prediction is a single realization of a future scenario chosen from many equally plausible ones [35]. Activity data were combined with emissions factors to estimate emissions from the projected land use scenarios under this “hard” model for the period 2015–2024.

Finally, we estimated the income that could potentially be generated by REDD+ projects targeting the end of land invasions by performing a back-of-the-envelope calculations based on historical carbon prices from voluntary markets using US$5/ton, a price used by many early REDD+ buyers in bilateral agreements [36, 37]. Projected emissions from deforestation due to land invasions for the period 2015–2024 were converted to 2015 US$ using net present values (NPV) at an 8% discount rate [38].

### Building an Indigenous perspective of REDD+ and territorial conflicts

#### Facilitating exchanges of viewpoints

To discuss if and how REDD+ could be an opportunity to advance Indigenous rights and conserve forests, 12 workshops and meetings (Table 1) were organized during the *Partnership Project*. Participants in these meetings included Indigenous traditional authorities, technicians, and NGOs. Some meetings addressed fundamental questions regarding the design and implementation of REDD+ in Indigenous territories, while others tackled specific topics, for example carbon ownership rights. The analysis presented in this paper was nourished by these discussions.

**Table 1:** REDD+ related workshops and meetings held with Indigenous authorities, technicians and communities during the *Partnership Project* (2010–2012).

***Informative sessions and thematic workshops for an organized indigenous civil society***	
Information Session on Climate Change and REDD+	26 participants /16 NGOs
Indigenous Subsistence, Conservation and REDD+	30 participants /11 NGOs
Carbon Property	20 participants /8 NGOs
***Training activities for traditional authorities***	
REDD+ Case Studies	77 participants
Benefits Sharing	44 participants
Safeguards and Certification	65 participants
International Climate Change Negotiation Simulation	29 participants
Carbon Property and Indigenous Safeguards within the REDD+ Framework	45 participants
Discussion with Indigenous Authorities about Draft Bill on Land Invasions	20 participants
***Traditional Authorities encounters for analysis and responses to REDD+***	
Nusagandi, Kuna Yala Comarca*	28 participants
Wichubuala, Kuna Yala Comarca	44 participants
Gaigirdub, Kuna Yala Comarca	40 participants

*Comarcas are large Indigenous territories established by law.

#### The legal context, carbon ownership rights and territorial conflicts

From the *Partnership Project’s* inception, it was clear that the REDD+ proposal could not move forward without evaluating the legal context of land and carbon ownership, and territorial conflict resolution. Clarity on carbon property is essential for the eventual implementation of a REDD+ scheme in Indigenous territories and elsewhere in the country. Co-author, AA, therefore compiled national laws on land property and forests, analyzed how Indigenous rights were recognized in different texts, and developed a comparative chart of laws for the Comarcas. Work also included the compilation of international law norms and jurisprudence of the Inter-American Human Rights Court on land property, territory, and natural resources.

## Results and discussion

### Land use change in the Bayano

Our participatory accuracy assessment of the Hansen et al. [27] maps of forest cover and forest cover change in the Upper Bayano Watershed shows a high overall accuracy of 84.9% and 85.1% for the years 2013 and 2014, respectively (Text B in S1 File). Both user and producer accuracy was above 80%, which meets the recommendation of Thomlinson et al. [39] of an overall accuracy of 85%, with no class less than 70%. As such, we consider that the Hansen et al. [27] maps are adequate to monitor deforestation in the watershed. A non-negligible advantage of using the Hansen et al. [27] maps for the Upper Bayano Watershed is that high quality spatial data are scarce for the region, particularly because pervasive cloud cover precludes the availability of extensive time series of remote sensing data. We noted, however, that the Hansen et al. [27] maps tend to overestimate forest areas in some zones due to the classification of short fallows, scrublands and non-forested riparian vegetation (less than 5 m tall) around Lake Bayano as forests (user accuracy of 80%) (Text B in S1 File). Therefore, we would recommend that other groups wanting to use the Hansen et al. [27] maps for the development of a reference level, as we did and explicated in our method section, also carry out an statistical analysis through ground truthing to evaluate local accuracy and potentiality of these data.

**Fig 4:**
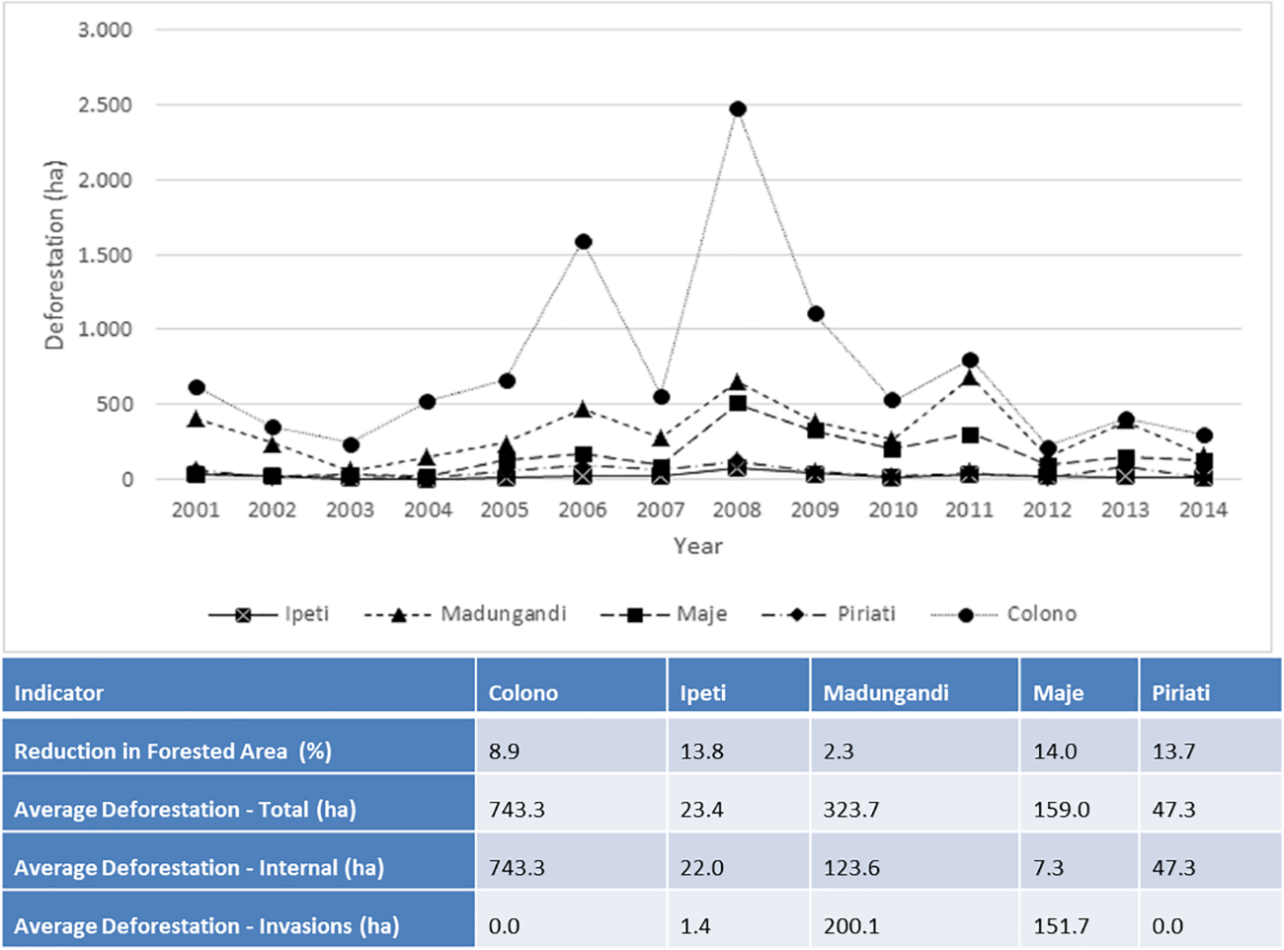
Total deforestation (ha) and indicators of deforestation (2001–2014) in Indigenous territories and colonist areas in the Upper Bayano Watershed.

After verifying the accuracy of the Hansen et al. [27] maps at the local scale, we analyzed differences in deforestation in Indigenous territories and non-Indigenous parts of the watershed. The areas with highest forest cover in 2001 correspond to two Indigenous territories, Madungandi and Majé, with 99.1% and 98.5%, respectively. Non-Indigenous areas conversely presented the lowest forest cover in the watershed, 74.2% in 2001. For the period 2001–2014, according to the Hansen et al. [27] maps, 17,901 ha of forests were cleared in the watershed, that is, 5.9% of the total forest area in 2001 (Table 2). During the same period, non-Indigenous lands lost 10,191 ha, accounting for ~57% of the total deforestation in the watershed. At 0.42% annual rate of forest loss, deforestation in the Upper Bayano Watershed is higher than Panama’s average of 0.35% for the period 2000–2012 [40] (Fig 4).

**Table 2:** Forest cover and forest cover changes (2001–2014) in Indigenous territories and non-Indigenous areas in the Upper Bayano Watershed.

Area	Year 2001(ha)	Year 2014 (ha)	Loss (ha)	Change (%)
**Non Indigenous**	85,327	75,136	10,191	12
**Ipeti**	2,804	2,353	451	16
**Madungandi**	196,204	191,689	4,515	2.3
**Majé**	15,643	13,419	2,224	14
**Piriati**	2,997	2,477	520	17
**Total**	**302,975**	**285,074**	**17,901**	**5.9**

In Indigenous territories, deforestation was categorized as internal (produced locally) or external (invasions) to calculate the effect of colonist invasions. Our analysis shows that invasions of Indigenous territories resulted in 5,006 ha of forest clearing (27.6% of the total deforestation) for the period of 2001–2014. The situation is of paramount concern in the Embera territory of Majé where 95.4% of total deforestation was caused by colonist invaders (Fig 4). In the Comarca Madungandi, colonist invasions account for 61.2% of the deforestation. Deforestation driven by invaders occurs in these two larger territories, particularly in non-inhabited forested zones, where monitoring and control by communities is more difficult. The situation is diametrically opposed in Ipeti where total deforestation produced by invaders is low (7%) and in Piriati where there is no deforestation associated to invaders. These two last territories are comparatively smaller in size, which along with strong local organizations, makes it more feasible to control access to land invaders. Most reductions in forest area in Ipeti and Piriati are produced locally (internal), mostly driven by subsistence concerns [41]. These results enrich discussions regarding the importance of tenure security as an essential tenet of REDD+ implementation, and stimulates the identification of ways to improve forest management governance and practice [6] since titling or granting legal rights are apparently insufficient to fully protect forests in Indigenous territories in Panama. Invasions in the Comarca Madungandi indeed accounted for more than half of total deforestation in the territory despite having been granted legal rights to this land 20 years ago.

**Fig 5:**
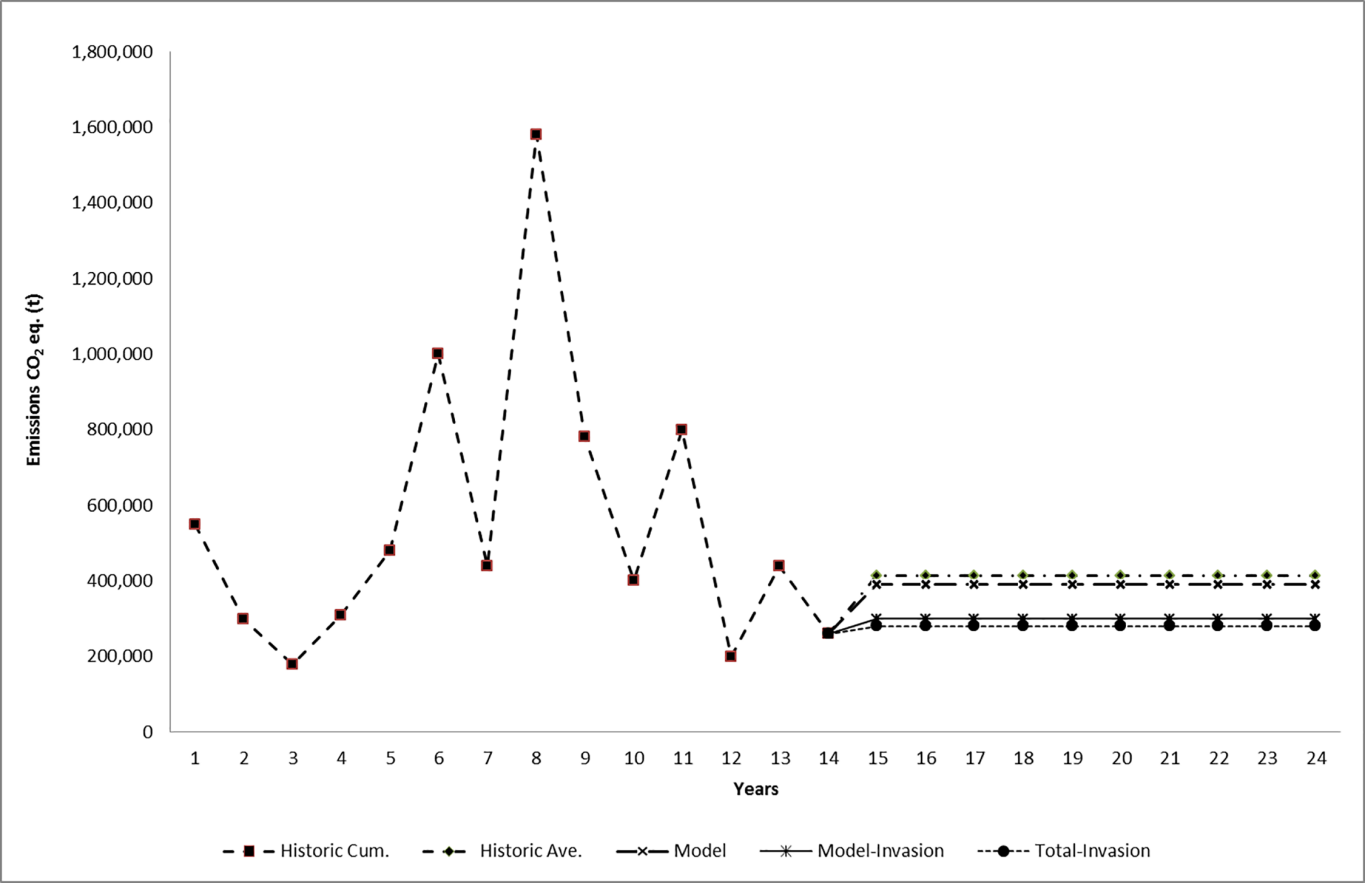
Historical and projected emissions of tCO2e in the Upper Bayano Watershed for 2001–2024.

Using the Terrset program, we projected deforestation for the period 2015–2024. Projected deforestation using a “hard” model estimated an average of 1,296 ha yr^-1^ of deforestation for the period 2015–2024. Projected deforestation in non-Indigenous areas, estimated at 743 ha yr^-1^, was higher than in Indigenous areas (553 ha yr^-1^). The model allowed us to project future forest lost due to invasions in Indigenous territories at 254 ha yr^-1^. We combined emissions factors with projected forest lost to develop emissions reference levels. The reference level computed by the “hard” model is slightly lower than the historical reference level, with both at ~400,000 tCO_2_e yr^-1^ (Fig 5). Invasions represented 25.2% of total carbon dioxide emissions in the watershed.

Our estimates assume that, in the wake of the 2014 ruling of the IACHR, the national government teamed up with Indigenous peoples to successfully halt invasions in Indigenous territories, and that Panama implemented REDD+ or a similar scheme of payment for ecosystem services. Using the “hard” model as a reference level, halting invasion could yield and income of US$3.3 million NPV between 2015 and 2024.

In more concrete terms, Gunas and Emberas have proposed a number of strategies to control deforestation due to land invasions on their territories including (i) creating, equipping and training an Indigenous “land protection ranger force” to patrol and protect their territories, (ii) reforesting with native species, and (iii) allowing degraded areas to recuperate naturally. These could be financed by hypothetical REDD+ income such as that estimated above. For example, a “land protection ranger force” would likely focus their efforts in patrolling the areas that have been deforested due to invasions, as well as vulnerable adjacent areas. Using the operating budget of Panama’s Barro Colorado Island Natural Monument (BCI)–with a comparable area of 5,400 ha–for control and vigilance actions as a proxy, the “land protection ranger force” would likely require ~US$201,000 yr^-1^ to carry out their activities, including salaries of 15 rangers, equipment, training, transportation, as well as maintenance and insurance of boats, vehicles and equipment (pers. comm. Mario Santamaría; Game Warden Supervisor, BCI). There would have to be an initial investment during the first year in vehicles, at least two, and one fully equipped boat, at an approximate total cost of $75,000. All of these costs are well within the projected annual income of US$330,000 that could be generated through REDD+ by avoiding forest loss from land invasions.

Reforesting with native tree species and letting the forest regrow have also been proposed as response to deforestation due to land invasions. Here again, hypothetical income from REDD+ might support such initiatives. Assuming a cost of ~US$563 yr^-1^ to reforest one hectare [42], approximately 586 ha of land could be restored annually in the watershed if all potential income generated from REDD+ (*i*.*e*. US$330,000) were to be invested in this activity. If funds were invested proportional to land area invaded for each territory (2001–2014), the Comarca Madungandi, and the Embera territories of Majé and Ipeti could replant 332, 252 and 2 ha yr^-1^, respectively. Ostrom [43], however suggested that bridging organizations that support community initiatives are a key element of the sustainable governance of common resources such as forests. In Ipeti and Piriati, bridging organizations indeed have played a fundamental role in supporting the two communities in REDD+ and PES initiatives [42, 44]. External support however is costly. If income generated by REDD+ were to serve to pay for such support, funds available for forest protection or reforestation would be much reduced. In this context, the Juma project in the Brazilian Amazon provides an interesting initial case study where the government invested, not only in direct payment for conservation, but also in education and health to support communities reduced impact on the forest [45].

### The legal context

An essential question emerging from our results on the importance of land-use invasions in Indigenous territories is: Who would receive the income from REDD+? In theory, benefits from carbon trading would be received by the “owner” of the carbon. Thus the concept of property rights central to define benefit sharing. The discussions that took place during the *Partnership project* highlighted that both the Indigenous and colonist sectors are concerned about the just distribution of potential REDD+ benefits and the lack of clarity regarding carbon ownership. Both sectors perceived the government’s lack of action in defining land and carbon rights as an obstacle to REDD+ implementation. The traditional authorities indicated unambiguously that Indigenous peoples in Panama, would request direct payment for REDD+, “Payment must be direct, without intermediaries, and in accordance to forest natural values: rivers, flora, fauna, etc.”

A review of national and Indigenous legislation related to carbon ownership and land invasions [41], as well as discussions held during the workshops of the *Partnership Project* examined several aspects of the question. In Panama, collective land property rights for Indigenous peoples are clearly recognized in the laws that created the five Comarcas and the law of Collective Lands (Law 72) [23]. As a consequence of their collective property, there is a wide spread sentiment amongst Indigenous peoples that they own the natural resources of their territories, including the trees and, hence, the carbon. Nevertheless, there is no explicit law establishing who owns carbon. Some relevant aspects of the legal context identified through this study are the following:

- Law 41 of 1998 (General Environmental Law) states in Article 79 [46]: “The State recognizes carbon capture as a forest environmental service and will establish a mechanism to capture financial and economic resources through jointly implemented programs, as internationally agreed.” In this context, it becomes critical to recall that customary rights establish that all natural resources within and Indigenous territory belong to that Indigenous group. The Inter-American Court of Human Right has issued decisions on land and natural resources rights, for example, in Nicaragua and Paraguay [47, 48]. These pronouncements could also be of use in Panama, as they state that Indigenous peoples own those rights. The Inter-American Human Rights Convention, in its Article 21, which stipulates that “Everyone has the right to the use and enjoyment of his property” and that “No one shall be deprived of his property except upon payment of just compensation…” could also be relevant [49].
- Law 1 of 1994 (Forest Law) states in Article 10 [50]: “The Forest State Patrimony is formed by all natural forests, lands upon which these forests are or were found…Forestry plantations established by the State on their own land will also form part of this patrimony”. This same Law in its Article 44 indicates: “Permits and concessions on forestry uses in Comarca areas or Indigenous Reserves and Communities will be authorized by INRENARE [now Ministry of Environment; MiAmbiente in Spanish], together with the respective Congresses, after a scientific management plan study is carried out”. According to existing legislation regarding forests, MiAmbiente facilitates the use of forestry patrimony on Indigenous communities through special permits, direct management, and concessions, as consecrated in Article 27 of the Forest Law and in accordance with Articles 101, 103 and 104 of the General Environmental Law (Law 41, 1 July 1998) [40]. It should be noted that the Executive Board Resolution No. 05–98, 22 January 1998 [51], presents special procedures for community permits in Indigenous areas.

The legal analysis raises concerns because rules differ according to the legislation and the internal norms of the territories (Table 3).

**Table 3:** Comparison of different Panamanian legislation regarding land and natural resources ownership, and natural resources management and conservation.

Question	Civil Code	Comarca Guna Yala	Panamanian Constitution	Comarca Embera Wounaan	Comarca Madungandi	Comarca Ngabe- Bugle	Comarca Wargandi	Collective Lands
Recognize private lands?	Yes	Those recognized by law	Yes	Those recognized by law	Those recognized by law (art. 21)	Those recognized by law	Recognize possessor right before the law	Those recognized by law
Recognize collective property?	No	Yes	Yes	Yes	Yes	Yes	Yes	Yes
Who is the owner of natural resources?	The owner	Indigenous peoples	The State and the franchiser	Indigenous	State and Indigenous peoples	Indigenous peoples/ art. 22 of the law	Indigenous peoples	Indigenous peoples?*
Who is in charge of resource management?	The owner	Indigenous peoples	State and owners	State and Indigenous peoples	Indigenous peoples	State with effective participation of Indigenous peoples	Indigenous peoples write down a management plan and MiAmbiente approves it	Indigenous peoples if these are in protected areas
Who is in charge of conservation?	The owner	Indigenous peoples	State and owners	State and Indigenous peoples	Indigenous peoples	State with effective participation of Indigenous peoples	Indigenous peoples and MiAmbiente	Indigenous peoples and MiAmbiente

*When “?” follows an answer, the legal context is unclear.

Discussions that took place during the 12 workshops supported by the *Partnership Project* complemented the legal analysis and highlight Indigenous peoples`claims for recognizing their positive role in forest conservation (Table 4) as illustrated by the following quotes:

“According to our vision, the indigenous people do not promote deforestation. What we cut is only for family subsistence. For colonists, their vision is to cut and burn large quantities of hectares. [Our] land use is compatible with REDD+.”

“One way or another, cutting and burning are partly responsible for gas emissions. But they are part of our way of living, and we are not going to change for anything in the World, not even with the best technologies. And it is a cycle, since when you stop for some time, it turns into forest again; for example, the Embera plant rice, corn; it is cultivated and harvested, afterwards the forest regenerate again…within its life cycle, [and it is] compatible with REDD+.”

**Table 4:** Consensus responses on REDD+-related questions discussed during the Partnership project workshops.

1. *How can a traditional Indigenous peoples’ vision of the forest (cultural value) be integrated into national policy for REDD+?*
- Compiling and systematizing the traditional vision of the forest (Cosmology), and disseminating it among Government Ministries. - Introducing the concept of traditional soil management. - Signing agreements between Indigenous peoples and ANAM [now Ministry of Environment; MiAmbiente in Spanish] for reciprocal training in conservation.
2. *How can the Indigenous peoples’ conservationist vision be explained*, *defended*, *and promoted where cattle has been seen for centuries as the only way to grow?*
-Explained: Sensitizing and implementing an educational workday on the importance of forests. -Defended: Emphasizing social aspects and elaborating environmental laws that are more flexible and sensitive to human needs. -Promoted: Having an effect on Government plans.
3. *What traditional use of the forests by Indigenous peoples (cut*, *and burn*, *and future land use) is compatible with REDD+?*
- Need to evaluate the following concepts: slash-and-burn, forests and traditional ceremonies, sacred and conservation refuge sites. - Need to evaluate the concept of community forest use proposed by ANAM.
4. *Can REDD+ help solve more important issues faced by Panama’s Indigenous peoples*, *such as land ownership?*
- We are still not clear if REDD+ can help solve the problem of land ownership because politics prevail.
5. *What are the conditions necessary to promote equitable collaboration between Indigenous peoples and the government*, *if REDD+ is implemented at the national level?*
- REDD+ directives must be clear, concise, and understandable. - ANAM [MiAmbiente] must “listen” to the Indigenous peoples and engage in a real “dialogue” with COONAPIP. - A technical cooperation agreement between both parties must be respected, monitored, and evaluated.
6. *What is REDD+’s main advantage for COONAPIP?*
- Its coherence with Indigenous cosmology (mother earth, the forests, land use, subsistence, conservation).
7. Who are the authorities that see a good opportunity in REDD+? Why?
-There is still too much suspicion.
8. *What does COONAPIP need to be positioned on REDD?*
- Dialogue with ANAM’s authorities. - Dialogue with sponsors of the World Bank Forest Carbon Partnership Facility (FCPC).

Opinions of workshop participants regarding REDD+ at the end of the process was divided between those who hoped that REDD+ could be an element contributing to their development and others with major concerns, in particular the prospects that REDD+ will (a) become another consulting gold-mine for foreigners; (b) strengthen MiAmbiente, and not Indigenous peoples; and (c) lead to expropriation of lands and territories. Key, therefore, to the development of REDD+ in countries with Indigenous territories is the true implementation of mechanisms for their full participation, and respect for Customary Laws, beyond lip service.

**Fig 6:**
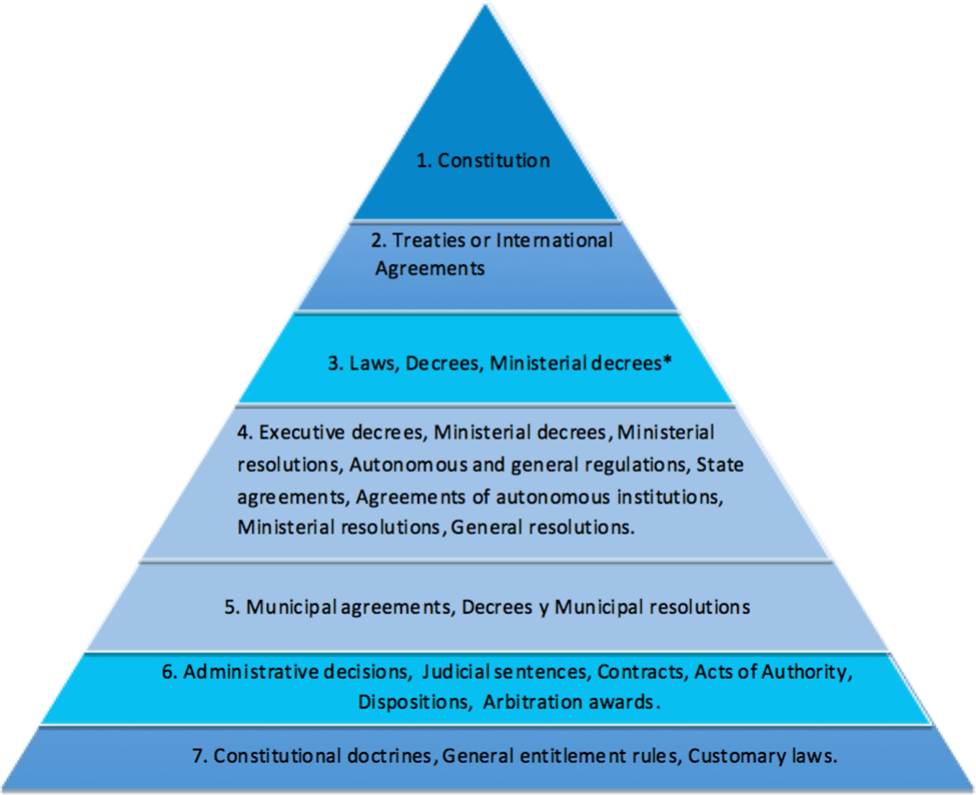
A diagram representing the hierarchy of legislation in the context of Panama.

To provide clarity and elucidate the preponderance of one law or norm over another one, we produced a variant of the Kelsen pyramid of law [52] (Fig 6) illustrating the hierarchy of legal norms in the context of Panama. It allowed all the partners to understand how, in the face of legal confusion or contradiction, one law or agreement could overrule another one. It also demonstrates that Customary Laws (*i*.*e*. Indigenous laws) fall at the bottom of the normative hierarchy.

The legal analysis further highlighted an important legislative gap regarding the invasion of Indigenous territories that is not considered in the penal code of Panama. For example, stealing a head of cattle warrants between 30 months and three years in jail. Thus, current legislation favours the traditional cultural values of cattle ranching but does not consider REDD+ realities. In response to this legal void, co-author AA, developed a draft bill (Text C in S1 File) inspired by discussions within the *Partnership Project*, which establishes a procedure for conducting evictions on invaded lands within Comarcas and Collective Lands, among other dispositions.

We suggest that the broad legal framework of Panama can explain the prevalence of land invasions in Indigenous territories. Like in many Latin American countries, the Panamanian Constitution (Article 123) indicates that the State will not tolerate the existence of land that is uncultivated, unproductive and idle (“incultas, improductivas u ociosas”), adding that the State will provide titles to those who make the land “productive” [41]. The pyramid of law (Fig 6) demonstrates that the Constitution is at the pinnacle of normative structures, taking precedence over all other levels in the hierarchy. Colonist farmers that invade forested Indigenous lands, therefore, consider that they are doing so in the context of their fundamental constitutional rights [53].

Thus, while REDD+ holds the opportunity to clarify carbon ownership, it may also be a liability since political good will is likely to be an essential element of future negotiations between the government and traditional Indigenous authorities on the topic. The wisdom of assuming political good will or the ability of States to control deforestation has been indeed singled out as an important barrier to REDD+ implementation [54, 55]. REDD+ readiness in Panama, as in other developing countries, therefore, demands resolving not only land tenure, but also the broader policy framework to remove perverse incentives when possible, including clarifying carbon ownership rights. There are avenues to resolve such problems. The Republic of Ecuador, for example, paved the way for fundamental shifts in legislation when, in 2008, they approved a new Constitution granting rights to nature. The application and real impact of those changes, however, remain to be evaluated [56]. Radical changes such as those made in Ecuador do not appear within reach in Panama in the short to middle term.

## Conclusions

Across Latin America Indigenous peoples own an extensive area of land [4]; 25.3% of the Amazon region is under Indigenous peoples’ custody [57] for example. Because Indigenous tenure has shown to be more efficient in controlling deforestation than private tenure [58, 13], we contend that Indigenous peoples should be considered essential partners of REDD+ and forest conservation programs and participatory methods offer a positive avenue to do so. In this context, and as demonstrated in this study, stemming the tide of land invasions is of paramount importance.

During the initial years of REDD+ implementation, interactions with Indigenous peoples were marred by accusations of procedural and ethical blunders [59, 60]. In Panama this resulted in fear and animosity toward the mechanism among some groups, in particular the Gunas [59]. We suggest that participatory studies can aid in modifying attitudes toward REDD+ by providing an opportunity to experience elements of the mechanism first-hand. One very clear outcome of the full engagement of Indigenous peoples in REDD+ MRV, as we did, is their enhanced sense of the value of the forest [22]. In the Upper Bayano Watershed, both the Guna and Embera opened up to the possibility of taking part in REDD+ at the end of this study, while remaining cautious and vigilant in discussions with UN-REDD and the Panamanian Ministry of the Environment.

Engaging in MRV, however, is only one element of the broader need to fully engage Indigenous peoples in REDD+. Our study demonstrates that the process might be fraught with legal impediments. During the *Partnership project*, traditional authorities and technicians agreed on the 13 pre-conditions necessary “before initiating the REDD+ program” (Text D in S1 File). These preconditions hinged around the recognition of land and Indigenous rights, capacity building and respect for Indigenous cosmology. The 13 recommendations formed the basis of a list of 19^th^ points central to the agreement between COONAPIP and the MiAmbiente to advance on REDD+ implementation [61]. How these points are translated into action will be the challenge of the next decade.

A key lesson learned throughout the *Partnership Project* is that territorial conflicts are present practically in every Indigenous territory. We, therefore, believe that REDD+ will need to count with a mechanism of conflict resolution, a topic that we address in companion papers [41, 53]. Mediation that would include relevant actors at the national and local scale, supported by an independent monitoring commission is one option to foster participation, in particular when the State is not actively interested/engaged in resolving the conflicts. As such, we offer the following blueprint as a series of steps that could generally guide REDD+ implementation in regions with unclear or poorly enforced land tenure.

- Phase I: identify relevant actors in REDD+, understanding their priorities and building capacity for REDD+.
- Phase II: identify conflicts, map them and assess their importance, develop alternative methods for conflict resolution.
- Phase III: analyze legislative texts to shed light on land rights and carbon ownership.
- Phase IV: build a consensus around the importance of providing better tools to resolve land-use conflicts and develop concrete proposals as our draft bill on land invasion (Text C in S1 File)

Of course, each case, community, nation and country has its own particularities. Solving the problem of land invasion in the context of REDD+ will therefore take somewhat of a different form depending of the context.

## Supporting information

S1 File Supplementary material. (DOCX)

S2 File Above ground biomass (Mg/ha) per plot in the study area. (XLSX)
