# Supplementary material for: A participatory approach to elucidate the consequences of land invasions on REDD+ initiatives: A case study with Indigenous communities in Panama
Source: PLoS One. 2017 Dec 19;12(12):e0189463. doi: 10.1371/journal.pone.0189463 (PMC5736191; doi:10.1371/journal.pone.0189463)
Supplement: S1 File — (DOCX) [file pone.0189463.s001.docx]

## **Supplementary Information**

**Text A. Participatory documentary filmmaking.**

Concerned by the unremitting tensions between Indigenous peoples and colonist farmers, as well as the perceived loss and degradation of forests in the watershed, both Indigenous and colonist community leaders sought funding with the NEL in 2012 to develop the collaborative project, *Juntos para Proteger Nuestra Cuenca* (*Together to Protect our Watershed*, in English). At the outset of the project, a common concern of all participants was their perceived disconnect or apathy by youth toward key issues that affect the region, including territorial disputes, deforestation, social tensions, lack of economic opportunities, and the loss of culture and traditions. With this in mind, the project set out to find a creative way to engage with the youth and settled on providing them with training on documentary filmmaking and related topics. Through this medium, youth were given a space and a voice to express their view of the situation in Upper Bayano Watershed and the issues that concern them. Over the course of the project, a total of eight workshops on film making were held in close collaboration with Wapikoni Mobile, a Canadian non-governmental organization specialized in training indigenous youth in video making. The 10 documentary films produced during the workshops reflect on the groups’ lives, culture, social dynamics and land-use, which are intimately tied to their forests and touched upon by the problem of land invasions.

Documentary available at http://www.wapikoni.ca/movies/aku-yala

**Text B. Hansen et al. [1] Forest Cover Map and Confusion Matrices**

The forest cover map by Hansen et al. [1] was launched by the World Resource Institute, through an initiative known as Global Forest Watch (http://www.globalforestwatch.org), to enable interactive, online forest monitoring with data covering more than a decade (2001-2014). These maps have a resolution of 30x30m. Previously, the highest resolution for global land cover and forest maps was 250x250m [2, 3].

Our accuracy assessment shows that degraded forests and growing vegetation affect classification accuracy, in particular in areas around the limit of 30% forest cover, i.e. a threshold used in many countries. The area of forests can be overestimated or underestimated depending on algorithm’s ability to discriminate forest from these other land uses. For 2013 and 2014, user accuracy for the water category was100% but producer accuracy was only 70%. Most misclassifications in the water category were related to inclusion of shallow waters, sand bars, and non-forested riparian vegetation (Table1 and 2).

Table 1. Confusion Matrix for the Hansen et al. [1] map of forest cover and forest cover change in 2013.

|  |  |  | **Reference** |  |  |  |
| --- | --- | --- | --- | --- | --- | --- |
|  |  | **Forest** | **Non-Forest** | **Water** | **Total** | **User´s Accuracy** |
|  | **Forest** | 64 | 8 | 8 | 80 | 80.0 |
|  | **Non-Forest** | 6 | 44 | 1 | 51 | 86.3 |
| **Map** | **Water** | 0 | 0 | 21 | 21 | 100.0 |
|  | **Total** | 70 | 52 | 30 | 152 | - |
|  | **Producer´s Accuracy** | 91.4 | 84.6 | 70.0 | Overall Accuracy = | 84.9 |

Table 2. Confusion Matrix for the Hansen et al. [1] map of forest cover and forest cover change in 2014

|  |  |  | **Reference** |  |  |  |
| --- | --- | --- | --- | --- | --- | --- |
|  |  | **Forest** | **Non-Forest** | **Water** | **Total** | **User´s Accuracy** |
|  | **Forest** | 64 | 8 | 8 | 80 | 80.0 |
|  | **Non-Forest** | 4 | 35 | 1 | 40 | 87.5 |
| **Map** | **Water** | 0 | 0 | 21 | 21 | 100.0 |
|  | **Total** | 68 | 43 | 30 | 141 | - |
|  | **Producer´s Accuracy** | 94.1 | 81.4 | 70.0 | Overall Accuracy = | 85.1 |

References

1. Hansen MC, Potapov PV, Moore R, Hancher M, Turubanova SA, Tyukavina A, et al. High-Resolution Global Maps of 21st-Century Forest Cover Change. Science. 2013; 342: 850–53. Available from: <http://earthenginepartners.appspot.com/science-2013-global-forest>.
2. Bicheron P, Defourny P, Brockmann C, Schouten L,  Vancutsem C,  Huc M,  Bontemps S, et al. GLOBCOVER Products Description and Validation Report. MEDIAS‐France. 2008. Available from: <http://publications.jrc.ec.europa.eu/repository/handle/JRC49240>
3. Hansen MC, Stehman SV, Potapov PV, Loveland TR, Townshend JRG,  DeFries RS, et al.  Humid tropical forest clearing from 2000 to 2005 quantified by using multitemporal and multiresolution remotely sensed data. Proc Natl Acad Sci U S A. 2008; 105(27): 9439– 9444.

**Text C. Draft bill on the resolution of territorial invasions.**

It is important to note that the draft bill was written in Spanish. The text of the articles is thus presented in both languages.

**Bill No. ____ of 201X By Which a Procedure to Evict For Land Invasions in Panama’s Comarcas, and Collective Indigenous Lands, and Other Dispositions are Established.**

**Justification**

Indigenous communities and collective lands in our country have suffered massive land invasions by colonists or people that do not belong to the Comarca, or collective lands. These invasions have affected natural resources on these Indigenous communities.

Invasions occur when people request land the State and see Indigenous territories as ideal places to expand the agricultural frontier. In some cases invasions are caused speculation land as land can be seized then sold.

No legal figure exist to that protects land neither the Comarcas nor the collective lands from invasions, since neither the law forming the Comarcas or those relevant to collective lands have legislated on this issue. Only collective rights ownership is recognized over their land, but there is no law that specifies what to do in case of land invasions.

In the same way, neither Law 58 of October 8^th^ 2010, that created the National Authority for Land Management, nor the actual Agrarian Code, regulate the resolution of Indigenous communities’ invasions.

Therefore, we present this bill to address this legal gap and in which legal authority is being provided to civil right municipal courts, to attend eviction for land invasions in the Comarcas, and collective lands. Such processes will follow the established indictment processes regulated by Panama’s Judicial Code.

This bill introduces alternative methods of conflict resolution by indicating that the Party can submit their differences to the Mediation Centers of the Judicial Organ before a Judge issues sentence, according to the established procedures for these Centers.

At present, there is no sanction on the Penal Code for invasions of Indigenous Comarcas, and Collective Lands land, therefore we propose prison sanction for those people dedicated to land invasions. On this sense we, submit to consideration of the National Deputy’s Assembly, this bill to become Law of the Republic.

**Article 1.** The objective of the present law is to establish a procedure to evict people who invade, occupy, or illegally usurp collective land property belonging to the Comarcas, and Indigenous Collective Lands./ *El objetivo de la presente ley es establecer un procedimiento para el lanzamiento de personas que invaden, ocupen o usurpen ilegalmente tierras de propiedad colectiva perteneciente a las Comarcas y tierras colectivas indígenas.*

**Article 2.** Authority is provided to municipal, or comarcal judges from the civil branch to address invasion, occupation, or illegal usurpation launching processes, that occur in the Comarcas, and Indigenous Collective Lands./ *Se le otorga competencia a los jueces municipales o comarcales del ramo civil para que conozcan de los procesos de lanzamiento por invasión, ocupación o usurpación ilegal que ocurran en las Comarcas y Tierras Colectivas indígenas.*

**Article 3.** In Indigenous Comarcas where there are no municipal, or comarcal judges; judges from the municipal courts in municipal district, to which the Comarca belonged before its creation will be competent./ *En las Comarcas Indígenas donde no haya jueces municipales o comarcales será competente para conocer este tipo de proceso el juzgado municipal del distrito municipal al que pertenecía dicha Comarca antes de su creación.*

**Article 4.** The eviction demands must be presented through a lawyer by traditional authorities from each of the invaded Comarcas, or collective lands. Power, and demand must comply with the requirements of the Judicial Code, and procedures submitted through an indictment process. / *La demanda de lanzamiento deberá ser presentado, mediante abogado, por las autoridades tradicionales de cada una de las Comarcas Invadidas o de la Tierras Colectivas. El poder y la demanda deberán cumplir los mismos requisitos que exige el Código Judicial, y el procedimiento estará sometido al proceso sumario.*

**Article 5.** The National Direction of Indigenous Policies of the Ministry of Government will be responsible, as requested by interested parts; to provide certification that establishes who are the traditional authorities from the Comarca, or Collective Lands./ *Le corresponderá a la Dirección Nacional de Política Indígena del Ministerio de Gobierno, a solicitud de la parte interesada, expedir la certificación en el que consten quienes son las autoridades tradicionales de la Comarca o de las Tierras Colectivas.*

**Article 6.** Before issuing sentence any Party may submit the land conflict to a Mediation Center of the Judicial Organ to be solved according the established procedures for these Centers./ *Antes de emitir sentencia cualquiera de las partes podrá someter el conflicto de tierras al Centro de Mediación del Órgano Judicial, y la misma será dirimida conforme al procedimiento establecido por dicho Centr*o.

**Article 7.** Therefore, we need to add a new article to the Penal Code./ *Agréguese un artículo nuevo al código penal así:*

**New Article:** Anyone who invades collective lands properties of a Comarca or Collective Land will be sanctioned with 4 to 6 years in prison. When groups that know that the lands are part of the Comarca, or Collective Land property promote invasions, the sanction will be from 5 to 7 years in prison.*/****Artículo Nuevo:*** *El que invada tierras de propiedad colectiva de una Comarca o de Tierras Colectivas será sancionado con 4 a 6 años de prisión. Cuando la invasión sea promovido por un grupo que se haya organizado a sabiendas de que las tierras forman parte de la propiedad colectiva de la Comarca o Tierras Colectivas la sanción será de 5 a 7 años de prisión.*

**Article 8**. This Law will be implemented when issued./*Esta Ley empezará a regir a partir de su promulgación.*

**Text D**. Pre-conditions that traditional authorities considered necessary “*before initiating the REDD+ program”* (Source: COONAPIP, Interim Report Packard Project, October 1^st,^ 2010)

1. Collective territorial rights on land, and natural resources value.

2. Promotion of treaties and international instruments on Indigenous Peoples Rights such as Agreement 169 from OIT, and United Nations Declaration on Indigenous Peoples Rights.

3. Elaboration of plans to build the capacities of Congresses and Indigenous Councils on management and functional aspects of forests and carbon.

4. Technical and professional training of indigenous people to be part of field personnel.

5. Review, analysis, and adjustment of existing national legislation on indigenous norms with regard to indigenous rights on natural resources, environment, biodiversity, forests, land, territories, oceans and pertinent issues.

6. Procure open and sincere discussion on the legal security of indigenous territories, collective lands, and comarcas.

7. Comply with the principle of free, previous, and informed consent using Indigenous Peoples own mechanisms.

8. Communication and coordination of all activities with COONAPIP.

9. Acknowledgement that the forests in indigenous territories are the collective property of that indigenous people.

10. Self-management of forests and forestry activities inside the territories.

11. Adoption of the concept “Buen vivir” and fair distribution of benefits.

12. Governance and management strengthening of Indigenous Peoples.

13. Protection of medicinal plants within forests on indigenous territories.
